# (*E*)-4-(1,3-Benzodioxol-5-yl)but-3-en-2-one

**DOI:** 10.1107/S1600536811004077

**Published:** 2011-02-09

**Authors:** S. Sarveswari, V. Vijayakumar, Priya Susan Mathew, Rafael Mendoza-Meroño, Santiago García-Granda

**Affiliations:** a Organic Chemistry Division, School of Advanced Sciences, VIT University, Vellore 632 014, India; b Departamento de Química Física y Analítica, Facultad de Química, Universidad de Oviedo – CINN, C/ Julián Clavería 8, 33006 Oviedo, Asturias, Spain

## Abstract

In the title compound, C_11_H_10_O_3_, the benzodioxole ring adopts a flattened [puckering parameters: *q*_2_ = 0.107 (2) Å, ϕ_2_ = 160 (1)°] envelope conformation with the methylene C atom as the flap. The crystal packing features chains, parallel to the *c* axis, composed of dimers connected by weak C—H–O hydrogen bonds and extending in layers in the *bc* plane.

## Related literature

For the synthesis of chalcones, see: Loh *et al.* (2010 ▶). For a related structure, see: Gao & Ng (2006 ▶).
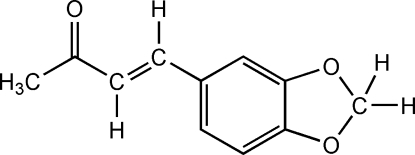


## Experimental

### 

#### Crystal data

C_11_H_10_O_3_
*M* *_r_* = 190.19
Monoclinic, 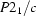

*a* = 5.3469 (3) Å
*b* = 16.4849 (8) Å
*c* = 10.5475 (6) Å
β = 99.183 (5)°
*V* = 917.77 (9) Å^3^
*Z* = 4
Cu *K*α radiation
μ = 0.83 mm^−1^
*T* = 293 K
0.18 × 0.11 × 0.09 mm

#### Data collection

Oxford Diffraction Xcalibur Ruby Gemini diffractometer
Absorption correction: multi-scan (*CrysAlis PRO*; Oxford Diffraction, 2009 ▶) *T*_min_ = 0.950, *T*_max_ = 1.000
5375 measured reflections
1737 independent reflections
1121 reflections with *I* > 2σ(*I*)
*R*_int_ = 0.029

#### Refinement

*R*[*F* ^2^ > 2σ(*F* ^2^)] = 0.036
*wR*(*F* ^2^) = 0.094
*S* = 0.92
1737 reflections
167 parameters
All H-atom parameters refined
Δρ_max_ = 0.12 e Å^−3^
Δρ_min_ = −0.15 e Å^−3^

### 

Data collection: *CrysAlis PRO* (Oxford Diffraction, 2009 ▶); cell refinement: *CrysAlis PRO*; data reduction: *CrysAlis PRO*; program(s) used to solve structure: *SIR92* (Altomare *et al.*, 1994 ▶); program(s) used to refine structure: *SHELXL97* (Sheldrick, 2008 ▶); molecular graphics: *ORTEP-3 for Windows* (Farrugia, 1997 ▶); software used to prepare material for publication: *WinGX* (Farrugia, 1999 ▶).

## Supplementary Material

Crystal structure: contains datablocks global, I. DOI: 10.1107/S1600536811004077/ng5110sup1.cif

Structure factors: contains datablocks I. DOI: 10.1107/S1600536811004077/ng5110Isup2.hkl

Additional supplementary materials:  crystallographic information; 3D view; checkCIF report

## Figures and Tables

**Table 1:** Hydrogen-bond geometry (Å, °)

*D*—H⋯*A*	*D*—H	H⋯*A*	*D*⋯*A*	*D*—H⋯*A*
C7—H7⋯O1^i^	0.95 (2)	2.56 (2)	3.489 (2)	166 (1)
C9—H9⋯O1^i^	0.93 (2)	2.60 (2)	3.517 (2)	171 (1)
C8—H8⋯O2^ii^	0.93 (2)	2.90 (2)	3.819 (2)	177 (1)

Symmetry codes: (i) 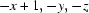; (ii) 
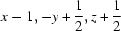
.

**Fig. 1.**
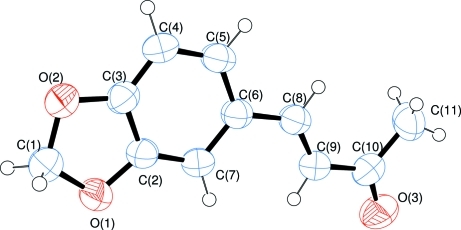
A view of (I), showing the atom-numbering scheme. Displacement ellipsoids are drawn at the 50% probability level.

**Fig. 2.**
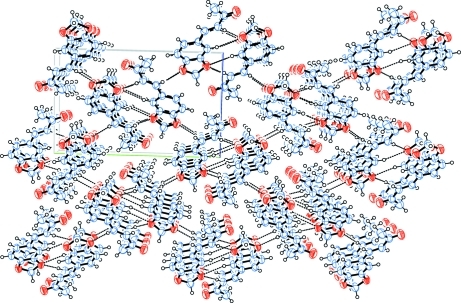
Packing diagram viewed parallel to the bc plane. Hydrogen bonds are indicated by dashed lines.

## supplementary crystallographic information

### Comment

Chalcones and its heterocyclic analogs have been used as intermediates in organic synthesis and exhibit diverse biological activities such as antimicrobial and cytotoxic agents. From a chemistry point of view, an important feature of chalcones and their heteroanalogs is the ability to act as activated unsaturated systems in conjugated addition reactions of carbanions. In continuation with our interest in the synthesis of chalcones (Loh *et al.*, 2010) herein we report the structure of the title compound (I).

In the title compound (I) the spatial arrangement of the keto group C(10)═ O(3) and the olefinic double bond C(8)═C(9) with respect to the single bond C9—C10 is *trans*, as indicated the C(8)—C(9)—C(10)—O(3) torsion angle value(-176.10 (18)°). The C(8)═C(9) (1.325 (2))Å), C(9)—C(10) (1.459 (2) Å) and C10═O3 (1.225 (2) Å) distances values are similar of the structures previously reported (Gao and Ng, 2006).

Plane A is refered to C(8)/C(9)/C(10)/O(3) atoms (maximum desviation C(9) 0.0229 (17) Å). The dihedral angle between C(2)/C(7) benzene ring (maximum desviation C(4) -0.0040 (18) Å) and *plane A* is 7.25 (10)°. In benzodioxole ring C(1) is displaced from mean plane by 0.1351 (22) Å, forming a flattened envelope conformation with C(1) as the flap atom. The packing in the crystal structure is dominated by molecular chains made of dimers connected by C—H–O weak hydrogen bonds and extended along *bc* plane.

Insert scheme 1.

The asymmetric unit consists of a single molecule (I), shown in Figure 1.

### Experimental

A mixture of acetone (3.0 g 0.005*M*) and benzo[*d*][1,3]dioxole-5-carbaldehyde (1.5 g 0.01*M*) and a catalytic amount of KOH in distilled ethanol was stirred for about 12 h, the resulting mixture was concentrated to remove ethanol then poured on to ice and neutralized with dill acetic acid. The resultant solid was filtered, dried and purified by column chromatography using 1:1 mixture of ethyl acetate and petroleum ether. Recrystallized from acetone; Yield: 49% and m.pt: 412–414 K.

### Refinement

At the end of the refinement the highest peak in the electron density was 0.124 e Å ^-3^, while the deepest hole was -0.154 e Å ^-3^.

### Crystal data

**Table:**

C_11_H_10_O_3_	*F*(000) = 400
*M**_r_* = 190.19	*D*_x_ = 1.376 Mg m^−^^3^
Monoclinic, *P*2_1_/*c*	Melting point: 413 K
Hall symbol: -P 2ybc	Cu *K*α radiation, λ = 1.54184 Å
*a* = 5.3469 (3) Å	Cell parameters from 1941 reflections
*b* = 16.4849 (8) Å	θ = 4.2–70.6°
*c* = 10.5475 (6) Å	µ = 0.83 mm^−^^1^
β = 99.183 (5)°	*T* = 293 K
*V* = 917.77 (9) Å^3^	Prismatic, yellow
*Z* = 4	0.18 × 0.11 × 0.09 mm

### Data collection

**Table:**

Oxford Diffraction Xcalibur Ruby Gemini diffractometer	1737 independent reflections
Radiation source: fine-focus sealed tube	1121 reflections with *I* > 2σ(*I*)
graphite	*R*_int_ = 0.029
Detector resolution: 10.2673 pixels mm^-1^	θ_max_ = 70.5°, θ_min_ = 5.0°
ω scans	*h* = −5→6
Absorption correction: multi-scan (*CrysAlis PRO*; Oxford Diffraction, 2009)	*k* = −14→19
*T*_min_ = 0.950, *T*_max_ = 1.000	*l* = −12→11
5375 measured reflections	

### Refinement

**Table:**

Refinement on *F*^2^	Primary atom site location: structure-invariant direct methods
Least-squares matrix: full	Secondary atom site location: difference Fourier map
*R*[*F*^2^ > 2σ(*F*^2^)] = 0.036	Hydrogen site location: inferred from neighbouring sites
*wR*(*F*^2^) = 0.094	All H-atom parameters refined
*S* = 0.92	*w* = 1/[σ^2^(*F*_o_^2^) + (0.0547*P*)^2^] where *P* = (*F*_o_^2^ + 2*F*_c_^2^)/3
1737 reflections	(Δ/σ)_max_ < 0.001
167 parameters	Δρ_max_ = 0.12 e Å^−^^3^
0 restraints	Δρ_min_ = −0.15 e Å^−^^3^

### Special details

**Table:**

Geometry. All e.s.d.'s (except the e.s.d. in the dihedral angle between two l.s. planes) are estimated using the full covariance matrix. The cell e.s.d.'s are taken into account individually in the estimation of e.s.d.'s in distances, angles and torsion angles; correlations between e.s.d.'s in cell parameters are only used when they are defined by crystal symmetry. An approximate (isotropic) treatment of cell e.s.d.'s is used for estimating e.s.d.'s involving l.s. planes.
Refinement. Refinement of *F*^2^ against ALL reflections. The weighted *R*-factor *wR* and goodness of fit *S* are based on *F*^2^, conventional *R*-factors *R* are based on *F*, with *F* set to zero for negative *F*^2^. The threshold expression of *F*^2^ > σ(*F*^2^) is used only for calculating *R*-factors(gt) *etc*. and is not relevant to the choice of reflections for refinement. *R*-factors based on *F*^2^ are statistically about twice as large as those based on *F*, and *R*- factors based on ALL data will be even larger.

### Fractional atomic coordinates and isotropic or equivalent isotropic displacement parameters (Å^2^)

**Table:**

	*x*	*y*	*z*	*U*_iso_*/*U*_eq_	
O2	0.5552 (2)	0.23540 (7)	−0.17522 (12)	0.0686 (3)	
O1	0.5790 (2)	0.09846 (7)	−0.13128 (11)	0.0669 (4)	
C2	0.4130 (3)	0.13463 (9)	−0.06159 (15)	0.0528 (4)	
C6	0.1246 (3)	0.14906 (9)	0.08521 (14)	0.0528 (4)	
C5	0.1132 (3)	0.23186 (10)	0.05728 (16)	0.0597 (4)	
C7	0.2809 (3)	0.09884 (10)	0.02303 (16)	0.0556 (4)	
C3	0.3994 (3)	0.21645 (9)	−0.08768 (15)	0.0557 (4)	
C8	−0.0220 (3)	0.11602 (10)	0.17895 (16)	0.0563 (4)	
C4	0.2514 (3)	0.26731 (10)	−0.02911 (17)	0.0627 (4)	
C9	−0.0146 (3)	0.04080 (10)	0.22361 (17)	0.0591 (4)	
O3	−0.1186 (3)	−0.06111 (8)	0.35856 (14)	0.0857 (4)	
C10	−0.1536 (3)	0.00895 (11)	0.32109 (16)	0.0632 (4)	
C11	−0.3362 (5)	0.06204 (16)	0.3750 (3)	0.0802 (6)	
C1	0.6491 (4)	0.16001 (11)	−0.2141 (2)	0.0683 (5)	
H8	−0.129 (3)	0.1522 (10)	0.2107 (17)	0.065 (5)*	
H7	0.295 (3)	0.0424 (10)	0.0405 (15)	0.058 (4)*	
H4	0.242 (3)	0.3245 (11)	−0.0461 (17)	0.074 (5)*	
H9	0.085 (3)	0.0018 (11)	0.1929 (15)	0.069 (5)*	
H5	0.007 (3)	0.2651 (10)	0.0987 (15)	0.061 (4)*	
H1A	0.567 (3)	0.1472 (11)	−0.304 (2)	0.079 (6)*	
H1B	0.839 (4)	0.1638 (11)	−0.2067 (17)	0.078 (5)*	
H11A	−0.415 (6)	0.0290 (19)	0.423 (3)	0.156 (12)*	
H11C	−0.247 (5)	0.1005 (18)	0.433 (3)	0.148 (12)*	
H11B	−0.442 (6)	0.0918 (19)	0.316 (3)	0.147 (12)*	

### Atomic displacement parameters (Å^2^)

**Table:**

	*U*^11^	*U*^22^	*U*^33^	*U*^12^	*U*^13^	*U*^23^
O2	0.0755 (7)	0.0532 (7)	0.0818 (8)	0.0007 (5)	0.0272 (6)	0.0100 (6)
O1	0.0752 (8)	0.0532 (7)	0.0789 (8)	0.0086 (5)	0.0323 (6)	0.0055 (6)
C2	0.0533 (8)	0.0466 (8)	0.0587 (9)	0.0033 (7)	0.0092 (7)	−0.0001 (7)
C6	0.0540 (8)	0.0482 (9)	0.0560 (9)	0.0025 (7)	0.0082 (7)	−0.0004 (7)
C5	0.0658 (10)	0.0485 (9)	0.0663 (10)	0.0081 (8)	0.0148 (8)	−0.0030 (8)
C7	0.0602 (9)	0.0430 (9)	0.0643 (10)	0.0042 (7)	0.0121 (7)	0.0024 (8)
C3	0.0569 (9)	0.0497 (9)	0.0605 (10)	−0.0023 (7)	0.0090 (7)	0.0046 (7)
C8	0.0565 (9)	0.0530 (10)	0.0594 (9)	0.0041 (7)	0.0094 (7)	−0.0033 (8)
C4	0.0719 (10)	0.0427 (9)	0.0745 (11)	0.0031 (8)	0.0144 (8)	0.0047 (8)
C9	0.0610 (9)	0.0497 (10)	0.0684 (10)	0.0019 (7)	0.0153 (8)	−0.0020 (8)
O3	0.1042 (10)	0.0586 (8)	0.0963 (10)	−0.0024 (7)	0.0216 (8)	0.0171 (7)
C10	0.0642 (10)	0.0564 (10)	0.0673 (10)	−0.0067 (8)	0.0048 (8)	0.0047 (8)
C11	0.0771 (13)	0.0829 (15)	0.0875 (16)	0.0063 (12)	0.0340 (12)	0.0146 (14)
C1	0.0753 (12)	0.0575 (10)	0.0766 (13)	0.0014 (9)	0.0260 (10)	0.0071 (9)

### Geometric parameters (Å, °)

**Table:**

O2—C3	1.3746 (19)	C8—C9	1.325 (2)
O2—C1	1.425 (2)	C8—H8	0.926 (18)
O1—C2	1.3755 (18)	C4—H4	0.960 (18)
O1—C1	1.428 (2)	C9—C10	1.459 (2)
C2—C7	1.358 (2)	C9—H9	0.927 (18)
C2—C3	1.376 (2)	O3—C10	1.225 (2)
C6—C5	1.396 (2)	C10—C11	1.490 (3)
C6—C7	1.410 (2)	C11—H11A	0.90 (3)
C6—C8	1.462 (2)	C11—H11C	0.95 (3)
C5—C4	1.390 (2)	C11—H11B	0.92 (3)
C5—H5	0.944 (17)	C1—H1A	1.005 (19)
C7—H7	0.948 (17)	C1—H1B	1.009 (19)
C3—C4	1.366 (2)		
			
C3—O2—C1	105.93 (12)	C3—C4—H4	122.5 (11)
C2—O1—C1	105.90 (12)	C5—C4—H4	121.1 (11)
C7—C2—O1	127.63 (14)	C8—C9—C10	126.69 (16)
C7—C2—C3	122.73 (14)	C8—C9—H9	120.7 (11)
O1—C2—C3	109.63 (13)	C10—C9—H9	112.6 (11)
C5—C6—C7	119.01 (14)	O3—C10—C9	119.83 (17)
C5—C6—C8	119.81 (14)	O3—C10—C11	120.43 (17)
C7—C6—C8	121.17 (14)	C9—C10—C11	119.74 (17)
C4—C5—C6	122.66 (16)	C10—C11—H11A	105 (2)
C4—C5—H5	118.7 (10)	C10—C11—H11C	110.3 (18)
C6—C5—H5	118.6 (10)	H11A—C11—H11C	106 (2)
C2—C7—C6	117.36 (15)	C10—C11—H11B	115.3 (19)
C2—C7—H7	121.4 (9)	H11A—C11—H11B	115 (3)
C6—C7—H7	121.3 (9)	H11C—C11—H11B	106 (3)
C4—C3—O2	128.40 (14)	O2—C1—O1	107.73 (14)
C4—C3—C2	121.79 (15)	O2—C1—H1A	109.7 (11)
O2—C3—C2	109.80 (13)	O1—C1—H1A	108.3 (11)
C9—C8—C6	126.90 (16)	O2—C1—H1B	108.7 (10)
C9—C8—H8	117.3 (11)	O1—C1—H1B	110.9 (10)
C6—C8—H8	115.8 (11)	H1A—C1—H1B	111.5 (15)
C3—C4—C5	116.44 (16)		
			
C1—O1—C2—C7	174.79 (18)	C7—C2—C3—O2	179.18 (15)
C1—O1—C2—C3	−6.33 (18)	O1—C2—C3—O2	0.24 (18)
C7—C6—C5—C4	−0.4 (3)	C5—C6—C8—C9	−173.77 (17)
C8—C6—C5—C4	178.81 (16)	C7—C6—C8—C9	5.4 (3)
O1—C2—C7—C6	178.83 (15)	O2—C3—C4—C5	−179.36 (16)
C3—C2—C7—C6	0.1 (2)	C2—C3—C4—C5	−0.6 (3)
C5—C6—C7—C2	0.0 (2)	C6—C5—C4—C3	0.7 (3)
C8—C6—C7—C2	−179.22 (15)	C6—C8—C9—C10	177.30 (16)
C1—O2—C3—C4	−175.19 (18)	C8—C9—C10—O3	−176.10 (17)
C1—O2—C3—C2	5.96 (19)	C8—C9—C10—C11	3.5 (3)
C7—C2—C3—C4	0.3 (3)	C3—O2—C1—O1	−9.8 (2)
O1—C2—C3—C4	−178.69 (15)	C2—O1—C1—O2	9.91 (19)

### Hydrogen-bond geometry (Å, °)

**Table:**

*D*—H···*A*	*D*—H	H···*A*	*D*···*A*	*D*—H···*A*
C7—H7···O1^i^	0.95 (2)	2.56 (2)	3.489 (2)	166 (1)
C9—H9···O1^i^	0.93 (2)	2.60 (2)	3.517 (2)	171 (1)
C8—H8···O2^ii^	0.93 (2)	2.90 (2)	3.819 (2)	177 (1)

Symmetry codes: (i) −*x*+1, −*y*, −*z*; (ii) *x*−1, −*y*+1/2, *z*+1/2.
